# Towards a nonlinear “neuroimmune gate-model”: beyond stellate ganglion block and vagus nerve stimulation

**DOI:** 10.3389/fnins.2026.1976225

**Published:** 2026-09-29

**Authors:** Lorenz Fischer, Hans-Georg Schaible

**Affiliations:** 1 Neural Therapy, University of Bern, Bern, Switzerland; 2 Institute of Physiology/Neurophysiology, Jena University Hospital, Friedrich-Schiller-University Jena, Jena, Germany

**Keywords:** neuroimmune gate model, neuroscience, nonlinearity, stellate ganglion block, vagus nerve stimulation

## Abstract

We propose a three-level neuroimmune gate model to characterize the communication between the nervous system and the immune system. The first gate, Gate 1, is the peripheral neuroimmune gate. Here, immune cells, inflammatory mediators, sensory neurons, autonomic nervous system neurons, blood vessels, and specific tissue components interact. The second gate, Gate 2, is the spinal neuroimmune gate and includes spinal afferents, spinal neurons and circuits, preganglionic sympathetic neurons, and microglia. The third gate, Gate 3, comprises supraspinal structures, including the nucleus tractus solitarii (NTS), the rostral ventrolateral medulla (RVLM), the locus coeruleus, the hypothalamus, and corticolimbic circuits. These three gates are functional centers that process and integrate inflammatory and neuronal signals dynamically and nonlinearly. This process can amplify or attenuate inflammatory reactions and disease states. The gates communicate with each other via neuronal pathways and humoral mediators. Sympathetic pro-inflammatory influences, vagal anti-inflammatory influences, and preexisting pro-inflammatory conditions (preload) are particularly important in all three gates. Unfavorable nonlinear interactions within and between these gates may cause tissue-threatening stimuli to evoke exaggerated responses, such as hyperinflammation. Understanding the three gates and their interactions can provide a conceptual framework for diagnosis and intervention. With this in mind, we will discuss the benefits of nonlinear therapeutic approaches, such as stellate ganglion blockade (SGB) and vagus nerve stimulation (VNS). We propose considering SGB and VNS as nonlinear therapeutic approaches with the capacity to close the proinflammatory neuroimmune gates based on endogenous regulatory mechanisms.

## 1 Introduction

It is becoming increasingly clear that the autonomic nervous system (ANS) and the immune system are coupled to form the neuroimmune system (Elenkov et al., 2000; Tracey, 2002). Within the ANS, the sympathetic nervous system (SNS) typically promotes inflammation, whereas the parasympathetic branch, primarily via the vagus nerve, combats it. The neuroimmune system responds to each internal or external stimulus — whether traumatic, physical, infectious, or psychological — according to the principles of homeostasis and economy. This response may involve physiological inflammation. Physiological balance is maintained as long as the ANS is in equilibrium. Dominant sympathetic activity may lead to hyperinflammatory responses and chronic persistent inflammation upon a new stimulus. This can also disturb microcirculation and elicit subsequent degeneration (Puente de la Vega Costa et al., 2016; Fischer et al., 2022; Jänig, 2022).

The neuroimmune system cannot be understood by examining its mechanisms in isolation because each pathological stimulus or therapeutic intervention influences the entire system, causing it to reorganize. Such reorganization is only possible in nonlinear, chaotic systems, as demonstrated by mathematical and physical theories (Mandelbrot, 1967; Nicolis and Prigogine, 1977). Similarly, we can use these theories to analyze physiological and pathophysiological events, as well as therapeutic effects such as stellate ganglion block (SGB) and vagus nerve stimulation (VNS) (Fischer et al., 2022). After short-term therapeutic interventions, the system can reorganize itself. Nonlinear effects in the neuroimmune system occur at three integrative levels, which we call “gates.”

In this short perspective article, we present a simplified three-gate model that could help us better qualify therapeutic methods, such as SGB and VNS; select appropriate combinations of approaches; and avoid unfavorable combinations of strategies. With further research, this initial model can be expanded and refined.

## 2 Proposal for a neuroimmune gate model

We consider the peripheral nervous system, spinal cord, and brain to be interdependent neuroimmune regulatory sites (gates) (Figure 1). Within each gate, neurons, immune cells, blood vessels, hormones, and mediators communicate with one another to determine the gate’s state. The gate opens if proinflammatory signals dominate, the gate closes, if inhibitory anti-inflammatory signals prevail.

**Figure 1:**
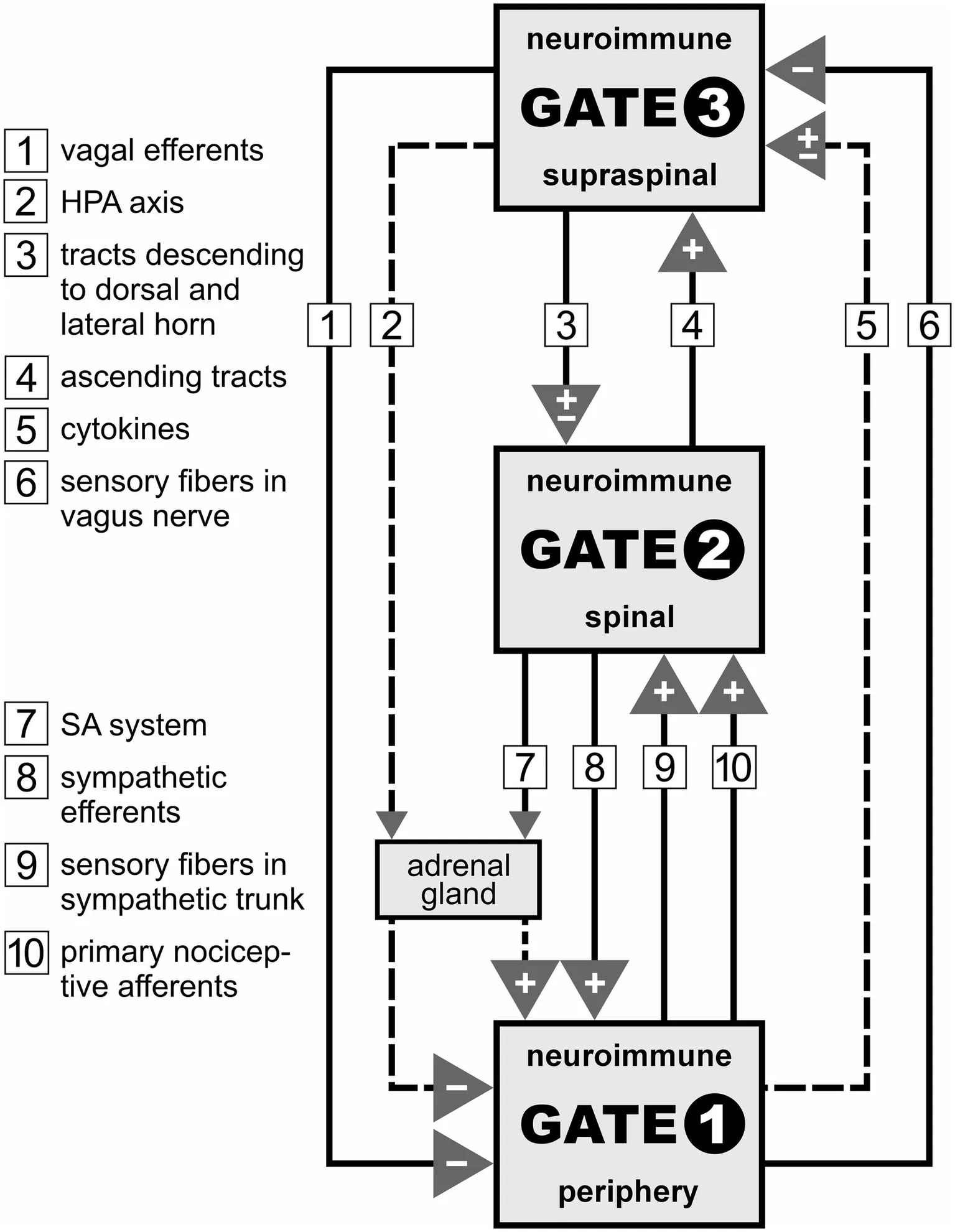
Schematic and simplified representation of the neuroimmune gates. The gates are not anatomically defined locations. They are a functional regulatory site where incoming signals are integrated in a context-dependent manner. Signals can be amplified by the gates (proinflammatory, indicated as + in the figure) or attenuated (anti-inflammatory, indicated as – in the figure). Proinflammatory indicates gate opening; antiinflammatory gate closing. This weighted output can be vagal, sympathetic, nociceptive or humoral. The figure suggests a binary open/closed state. However, this process is gradual. Furthermore, communication within the gates is largely nonlinear, involving positive feedback loops. This poses on the one hand a risk of hyperinflammation; on the other hand, it also presents an opportunity for nonlinear therapies such as SGB or VNS. These affect various gates directly or indirectly; see text.

The SNS, the vagus nerve, the hypothalamic–pituitary–adrenal (HPA-) axis, and cytokines do not act as persistent “gate openers” or “gate closers.” Rather, the actual state of these systems and the biological context determine whether they open or close the gates.

At each gate signals can be amplified or dampened. We believe that communication within the gates is characterized by nonlinear aspects (Fischer et al., 2022). For this reason, we present the following definitions.

### 2.1 Non-linear aspects of neuroimmune communication

When nonlinear dynamics and chaos theory are applied to nature and biology, they offer a more accurate representation of complex systems than linear methods (Mandelbrot, 1967; Lorenz, 1972; Nicolis and Prigogine, 1977; Fischer, 2017). In this short perspective article, we focus on a few important principles that we consider essential for neuroimmune communication within and between gates.

Non-linearity. A system’s output is not proportional to its input. Nonlinearity is a prerequisite for self-organization.

Positive feedback loops. An effect amplifies its own cause and may generate a different state. This makes physiological sense in the defense of microorganisms, for example. However, it can also rapidly deteriorate, causing hyperinflammation or, depending on other factors, chronic inflammation.

A small stimulus with a large effect. This is possible due to non-linear reactions and positive feedback, which amplify the initial stimulus. This applies to physiological, pathophysiological, and therapeutic stimuli, such as SGB and VNS.

Pre-exisiting conditions (preload, priming). In general, minimal differences in the system’s actual state can lead to completely different results when a new stimulus arrives, even at the same stimulus strength. This principle also applies to the neuroimmune system.

Capacity for self-organization. Nonlinear systems with positive feedback are unstable. However, they can adapt quickly due to their capacity for self-organization, affecting the entire system. The same applies, e.g., to SGB and VNS.

### 2.2 The three-gate model for the neuroimmune system

At each gate the nervous and immune systems communicate intensely, bidirectionally, and nonlinearly. The result can be either pro-or anti-inflammatory, and open or close the gate. This result is communicated to the other gates. All three gates together define the strength of pro-or anti-inflammation.

As early as Melzack and Wall (1965) demonstrated that spinal cord neurons do not simply respond to stimuli. Rather, they integrate inputs from the periphery, the spinal cord, and the brain. Similarly, this principle may apply to neuroimmune gates. The vagus nerve may close the “inflammatory gate” (VNS amplifies this effect). The SNS may open it, thus having a proinflammatory effect. Therefore, resetting the SNS with local anesthetics closes the “inflammatory gate.” However, the outcome of sympathetic immunomodulation depends on the organ and disease phase (Pfister and Fischer, 2009; Schaible and Straub, 2014; Strong et al., 2018). The SNS can exert anti-inflammatory effects in late disease states. For didactic reasons, we do not consider that communication within the gates may continuously change.

#### 2.2.1 Gate 1: the peripheral neuroimmune gate

The constituents of this gate include damaged tissue, the endothelium and microcirculation, immune cells, nociceptive and visceral afferents, vagal efferents (see below) and sympathetic efferents, and mediators such as neurotransmitters and cytokines. When signals arise from infection, injury, or disturbed microcirculation, immune mechanisms are activated, with extensive production of pro-inflammatory cytokines, chemokines, prostaglandins, ATP, and other mediators, as well as an increase in sensory neuronal excitability. A moderate “normal “opening of Gate 1 allows minor stimuli to be processed without the participation of the other gates. However, inflammation can become hyperinflammatory if the initial stimulus is too intense or if other unfavorable factors from the other gates influence it.

An inflammatory stimulus may elicit positive feedback loops with nonlinear dynamics (Fischer et al., 2022). Since neurons express receptors for inflammatory mediators (Schaible, 2014; Ji et al., 2016), substances such as substance P or cytokines can activate them. This increases the synthesis and release of substance P or cytokines (Galic et al., 2012). Substance P reinforces this positive feedback loop by stimulating the production of pro-inflammatory cytokines in macrophages (Bill et al., 1979). The influence of the SNS is intensified by adrenoceptors in immune cells (Bellinger and Lorton, 2014), which induce enhanced production of pro-inflammatory cytokines. Additionally, some cytokines enhance substance P synthesis in sympathetic ganglia (Freidin and Kessler, 1991). The integration of neuronal and chemical signals opens Gate 1. Gate 1 then communicates with Gates 2 and 3 via neuronal signals from sensitized nociceptors and vagal afferent fibers, as well as circulating cytokines (Chiu et al., 2013).

The following heuristic “equation” describes a simple threshold model for opening an inflammatory or neuroimmune gate, but it has no predictive quantitative value:


$${\mathrm{G1}}_{\text{inflamm}}=(D+N+P+\mathrm{Sa}-V-H)+$$

G1_inflamm_: activation of neuroimmune gate 1 (proinflammatory).

D: danger, such as tissue damage or viral infection.

N: nociception and nociceptive sensitization.

P: preload (pre-existing conditions or long-lasting load on the neuroimmune system from previous events).

Sa: the reinforcing effect of sympathetic activation.

V: vagal counterregulation.

H: HPA-mediated counterregulation.

If G1_inflamm_ exceeds the activation threshold, the gate opens. If P is high, lower magnitudes of D, N, or Sa are sufficient to exceed the threshold.

Notably, the vagus nerve innervates directly only the abdominal organs, not most somatic tissues. However, it can influence cholinergic immune cells with the α7 nicotinic acetylcholine receptor (α7nAChR) at various sites (e.g., the spleen; see Fujii et al., 2017 for details) and mediate anti-inflammatory effects in all organs by downregulating their proinflammatory activity (Berthoud and Neuhuber, 2019; Fujii et al., 2017).

#### 2.2.2 Gate 2: the spinal neuroimmune gate

Gate 2 encompasses segmental processing in the spinal cord, particularly in the dorsal horn. At this site, nociceptive afferents target excitatory and inhibitory interneurons, as well as ascending neurons, microglia, and descending neuronal pathways (Schaible et al., 2024). Spinal cord neurons either activate the brainstem, thalamus, amygdala, and higher supraspinal networks, including the limbic system, and/or they activate the ventral horn (the motor system) and the lateral horn (preganglionic sympathetic neurons), creating peripheral-spinal reflex arcs. Within spinal segments, interneurons connect the skin, musculoskeletal system, and internal organs. Segmental pain and inflammation can activate the dorsal horn via a positive feedback loop (Fischer, 2025). “Short circuits” between the SNS and nociceptive fibers can increase pain and inflammation (Jänig, 2022). The sensitization of spinal cord and sympathetic ganglion neurons increases the strength of positive feedback loops, and a small stimulus can also have a significant impact on pain and inflammation at this site. Negative emotions from gate 3 can reinforce processes in gate 2. Additionally, preganglionic sympathetic efferents reach the adrenal medulla and are modulated by the RVLM from gate 3. The SNS integrates all three gates but is mainly part of gate 2.

Microglia plays an important role in gate 2. The priming of microglia may determine the preload. A simple equation of the activation of gate 2 is:


$${\mathrm{G2}}_{\text{inflamm}}=(N+\mathrm{G1}+\mathrm{Sa}+\text{Mdesc})+$$

M desc: net effect of descending modulation from gate 3 (can be positive or negative).

#### 2.2.3 Gate 3: the supraspinal neuroimmune gate

Gate 3 is an autonomous neuroendocrine gate that regulates neuroimmune processes and pain. It encompasses the vagal and sympathetic brainstem nuclei, the hypothalamus, limbic structures, and cortical regions, such as the insula. These circuits integrate information from gates 1 and 2 and generate vegetative, neuroimmunological, and behavioral responses. These responses are transmitted via vagal and sympathetic efferent fibers to the hypothalamic–pituitary–adrenal (HPA-) axis and the SNS.

An important component of gate 3 is the nucleus tractus solitarii (NTS), which receives input from the vagus nerve. The NTS has connections to the vagal efferents and the SNS via the RVLM. The RVLM influences preganglionic sympathetic neurons and spinal cord neurons via descending fibers. The noradrenergic locus coeruleus and the reticular formation also modulate stress by processing viscero-nociceptive information. The hypothalamus integrates input from the NTS, other brainstem nuclei, the limbic system, and the cortex. The HPA-axis can combat inflammation via cortisol. The limbic system influences the neuroimmune system and pain processing via emotions. After complex integration, gate 3 informs gates 1 and 2 via three main feedback pathways: the vagal cholinergic anti-inflammatory feedback, sympathetic modulation of the neuroimmune system (including microcirculation), and the HPA-axis, which exerts anti-inflammatory effects on gate 1 (Jänig, 2022).

A simple equation for activation of gate 3 is:


$${\mathrm{G3}}_{\text{inflamm}}=(\mathrm{G1}+\mathrm{G2}+P+\text{Sstr}-\text{Sinh}-V)+$$

Sstr: supraspinal activation by stress and limbic reinforcement.

Sinh: supraspinal inhibitory control (e.g., by hypothalamus).

V: vagal counteraction.

#### 2.2.4 Preexisting conditions (preload, priming) affect the balance in all three gates

Several authors have demonstrated the importance of preload, referring to it as the “interference field” (Huneke, 1928), the “first strike” (Speranski, 1950), the “shifted setpoint” (Tracey, 2009), or the “neuromodulatory trigger” (Engel et al., 2022). Preload is important for all gates; however, priming is only necessary for gates 2 and 3. After priming, microglia respond excessively to new stimuli (Perry and Holmes, 2014; Niraula et al., 2017; Schaible et al., 2024). Preexisting conditions such as chronic stress, metabolic syndrome, abdominal diseases, and neuromodulatory triggers, such as periodontitis (Shiratori Tusita and Fischer, 2023) cause an imbalance in the ANS with sympathetic hyperactivity. Under these conditions, we believe that all three gates open more readily, respond more vigorously, and return more slowly to the homeostatic state. The hypothalamus may regulate this sympathetic overactivity. Preexisting conditions alone do not necessarily cause overt disease. However, an additional stimulus, such as an infection or stress, can trigger overactivity. The “sympathetic storm” can trigger a “cytokine storm,” which causes more damage than the viruses themselves, including endothelial damage, coagulopathy, and disturbed microcirculation (Fischer et al., 2022). Thus, preload puts the normal protective function of the neuroimmune reaction at risk (Fischer et al., 2022). Since the nonlinear system organizes itself using positive feedback loops, it is difficult to stop. We hypothesize that such positive feedback loops are also a putative mechanism of autoimmune diseases (Fischer et al., 2022).

If sympathetic activity is continuously enhanced, the neuroimmune system can adopt a state characteristic of chronic inflammation, associated with neuroplasticity in the central nervous system (Jänig, 2022) and the sympathetic ganglia (Alkadhi et al., 2005). Then even “physiological activity” can cause pathological responses. The persistent inflammation contributes to preload during aging and may cause a persistently enhanced sympathetic tone.

A simple equation for all gates is:


$$G_{\text{inflammation}}=(D+N+\mathrm{Sa}+P-V-H)+$$

## 3 Therapeutic perspectives

### 3.1 A non-linear therapeutic perspective

From our conceptual viewpoint, we consider the neuroimmune system to be primarily nonlinear; therefore, a suitable therapeutic approach should also be nonlinear. Such approaches include SGB and VNS. These therapies function as a “reset” (SGB) or a “stimulus” (VNS), allowing the system to reorganize itself in relation to homeostasis and economy. Therefore, we refer to these therapies as “nonlinear therapies.” One example of their potential is the reduction of pro-inflammatory cytokines following SGB (Liu et al., 2013; Yang et al., 2015; Chen et al., 2018).

Many diseases and dysregulations are caused by dysbalance in the ANS and misregulation in the neuroimmune system. Then even minor infections or psychic traumas may cause the system to overreact and evoke inflammatory states beyond physiological limits via cytokines (Fischer et al., 2022). Therefore, it is logical to attenuate the pro-inflammatory sympathetic tone (via SGB) and/or enhance anti-inflammatory vagal activity. The following paragraphs will describe the effects of these therapies on the three gates.

The awareness of the three gates helps in general to determine which therapies affect which gates. For example, a change in diet may affect gate 1, while psychotherapy may affect gate 3.

### 3.2 Stellate ganglion block and vagus nerve stimulation

For SGB, a local anesthetic is injected into the stellate ganglion of the cervical sympathetic trunk. For VNS, the vagus nerve is stimulated with electric impulses (Borovikova et al., 2000; Pavlov et al., 2020), which activate afferent vagal pathways to the NTS. This can influence the autonomic, neuroendocrine, and neuroimmune regulation.

#### 3.2.1 Effects on gate 1

SGB. It temporarily interrupts the positive feedback loops formed by nerve endings and immune cells. This interruption by a local anesthetic allows for reorganization. The net result is a reduction in pro-inflammatory cytokines and an increase in anti-inflammatory cytokines. Additionally, endothelial function and microcirculation improve, and sympathetically maintained nociceptor sensitization decreases (Fischer et al., 2022).

VNS. It activates cholinergic receptors in immune cells, which are part of the anti-inflammatory system, and reduces the production of pro-inflammatory cytokines (Tracey et al., 2002).

#### 3.2.2 Effects on gate 2

SGB. It can desensitize the immediate functional reflex connection between sympathetic nerve fibers and nociceptive afferents at the spinal segmental level. It can also reduce neuroplastic changes in the ganglion itself, hypothetically (Alkadhi et al., 2005).

VNS. No direct effect (only indirect through influencing RVLM).

#### 3.2.3 Effect of gate 3

SGB. It dampens the activity of neuronal circuits involved in stress and pain and thereby affects the hypothalamus (HPA-axis) and the RVLM.

VNS. It activates vagal afferents to the NTS, influencing neuronal circuits in the brainstem, hypothalamus, and HPA-related networks through this pathway. It can also attenuate the activity of sympathetic centers and support the parasympathetic counterregulation.

### 3.3 Synergies between SGB and VNS

The combined effects of SGB and VNS reduce pro-inflammatory sympathetic dominance, inhibit inflammation, and reduce pain. Both SGB and VNS, as well as their combination, may help to restore the neuroimmune balance and control pre-existing conditions thus allowing self-reorganization towards homeostasis (Fischer et al., 2022).

### 3.4 When to use SGB and when to use VNS?

In principle, both methods can be used for the same indications and different inflammatory and pain states because both SGB and VNS target autonomic balance, which can induce persistent effects through a “learning process.” However, SGB is more effective in cases of acute hyperinflammation. For chronic inflammation and pain, the effect of VNS may be equal. Importantly, our detailed analysis of the stellate ganglion’s anatomy and its connections to the vagal nerve (Puente Puente de la Vega Costa et al., 2016) suggests that electrode placement at the cervical site may prevent “pure” VNS. Hopefully, a more sophisticated VNS technique will minimize the costimulation of the sympathetic system. Stimulation at the ear may represent a more “pure” VNS stimulation.

For acute or recurring inflammation, SGB and VNS could be combined. The SGB could initially reset sympathetic activity, and the VNS could maintain this state to prevent new inflammatory episodes, e.g., in autoimmune diseases.

### 3.5 Prevention

We hypothesize that humans accumulate preloads throughout their lives. Perhaps the weakening of the immune system in older adults is partially caused by these preloads. We also hypothesize that preloads can be counteracted by SGB and VNS. Unlike suppressive therapies, these regulatory therapies follow a “non-linear” logic. Unlike linear drug therapy, they have almost no side effects. Conceptually, we see no reason why these treatments could not be used periodically throughout life to prevent or reduce the severity of diseases caused by neuroimmune dysregulation. However, SGB is invasive, and complications can occur. Invasiveness, limitations, and contraindications must be considered. Moreover, long-term studies are necessary to prove their preventive effect.

### 3.6 Practicability

VNS has more advantages than SGB. SGB is invasive with contraindications and can only be performed by highly specialized experts. In contrast, VNS is easy to use, even for patients at home. However, importantly, SGB has advantages in cases of acute and severe inflammation.

## 4 Conclusion

Our three-gate model does not claim to fully characterize the neuroimmune networks anatomically or physiologically. Rather, it should provide a didactic model to improve understanding of the increasingly complicated mechanisms of cooperation between peripheral tissue, the spinal cord, and the brain. The three-gate model can serve as a checklist to determine if therapies targeting gates 1, 2, and 3 can be combined to improve outcomes. We would like to emphasize the largely nonlinear and dynamic communication. Therefore, we propose conceptually that SGB and VNS, as nonlinear therapies, should be used and studied for their effects more often in the future. Because they aim to regulate rather than suppress and have only minor side effects.

## Data Availability

The original contributions presented in the study are included in the article/supplementary material, further inquiries can be directed to the corresponding author.
